# Analysis of diagnostic criteria for ECMO-associated pneumonia

**DOI:** 10.1017/ice.2024.181

**Published:** 2025-01

**Authors:** Julie England, Rachael Lee, Tammy Marshall, Rongbing Xie, Peggy Blood, Keith Wille, Enrique Gongora, Anoma Nellore, James K. Kirklin, Jeremey Walker

**Affiliations:** 1 Department of Internal Medicine, Division of General Internal Medicine, University of Alabama at Birmingham, Birmingham, AL, USA; 2 Department of Internal Medicine, Division of Infectious Diseases, University of Alabama at Birmingham, Birmingham, AL, USA; 3 Department of Internal Medicine, Division of Pulmonary, Allergy, and Critical Care Medicine, University of Alabama at Birmingham, Birmingham, AL, USA; 4 Department of Surgery, Division of Cardiothoracic Surgery, University of Alabama at Birmingham, Birmingham, AL, USA

## Abstract

Ventilator-associated pneumonia (VAP) is a well-established cause of morbidity in critically ill patients. Current VAP criteria exclude patients on extracorporeal membrane oxygenation (ECMO). This retrospective analysis tests the validity of VAP in this population, as well as a new proposed diagnostic criterion for ECMO-associated pneumonia.

## Introduction

Ventilator-associated pneumonia (VAP) is a well-established cause of morbidity and mortality.^1,2^ Current VAP research and National Health Safety Network (NHSN) definitions exclude patients on extracorporeal membrane oxygenation (ECMO).^3^ As use of ECMO grows,^4^ we must recognize and prevent pneumonia in this population. A meta-analysis reported ECMO-associated pneumonia occurred in 24.4 cases/1000 ECMO days,^2^ and smaller studies report an incidence of 55-74% in ECMO patients.^1^ Without an agreed-upon definition, many studies used diagnostic criteria for VAP to approximate pneumonia in ECMO.

Traditional diagnostic criteria for VAP may be unreliable in ECMO patients.^5^ First, they ignore important ECMO parameters: flow and sweep. During ECMO, blood is removed from the body, oxygenated and ventilated, then returned to the patient. To improve oxygenation, ECMO flow is increased. To improve ventilation, ECMO sweep is increased.^6,7^ Changes in these are important indicators of oxygenation and ventilation. Additionally, there is movement towards early weaning from mechanical ventilation for patients on ECMO,^4,8^ making diagnostic criteria for possible ventilator-associated pneumonia (PVAP), such as FiO_2_ and PEEP values, obsolete.^3^ Traditional signs of infection may be unreliable, as the extracorporeal circulation can mask fever and confound systemic inflammatory markers.^1,10^ Finally, chest radiography, incorporated in some definitions,^9^ may be subjective and limited in patients with acute respiratory distress syndrome (ARDS) or chest trauma requiring VV ECMO.

We must consider new diagnostic criteria for ECMO-associated pneumonia (EAP) to promote quality, document outcomes, and allow research. In this study, we perform a retrospective analysis to assess current NHSN PVAP definitions as diagnostic criteria for pneumonia in ECMO. We propose new EAP criteria, incorporating inflammatory, microbiologic, and ECMO data. We then test the criteria’s ability to diagnose and predict pneumonia in a clinically adjudicated cohort of ECMO patients with pneumonia.

## Methods

91 positive bronchoalveolar lavage (BAL) cultures from 52 ECMO patients between 2018 and 2021 were abstracted and reviewed for evidence of clinical pneumonia (CP). Cases were excluded when the organism does not typically cause pneumonia (*Candida* spp, *Enterococcus* spp), organism attributed to colonization, there is no documented antibiotic change, evidence of a severe concurrent non-pulmonary infection, a respiratory culture obtained <48 hours or >30 days after the ECMO starts, or the patient immunocompromised prior to cannulation. This led to the exclusion of 66 cultures from 32 patients. Included patients had positive BAL culture, concern for CP documented prospectively by the primary team, and antibiotic change reflective of diagnosis. Importantly, all cases were manually adjudicated by two authors to ensure agreement (JE, JW, RL). We considered the duration of active infection to be 5 days, with day 1 being the culture date.

Our proposed diagnostic criteria for EAP is build upon the established guidelines for PVAP but include parameters unique to ECMO populations. These criteria were developed based on literature review and clinical experience with ECMO patients.

In a patient on ECMO for ≥ 48 hours:

1. Worsening oxygenation, as evidenced by increase in daily minimum values of flow ≥ 10% in 24 hours or sweep ≥ 20% in 24 hours.
2. Evidence of inflammation or infection, demonstrated by temperature <36˚ C or >38˚C or leukocyte count <4k or >12k.
3. Positive culture data, including broncho-alveolar lavage (BAL) culture with >10^5^ CFU or Endotracheal aspirate culture with >10^4^ CFU of a pneumonia-causing organism.

Patients meeting criteria 1 + 2 were designated “Possible- EAP – Definition 1.” This definition excludes microbiological data. Patients with either criteria 1 + 3 or criteria 2 + 3 were designated “Possible EAP – Definition 2.” This definition includes microbiological data. Patients meeting all 3 criteria were designated “Confirmed EAP.”

Given the diverse causes of severe illness precipitating need for ECMO, each patient was used as their own control by reviewing all ECMO days post cannulation from day 3–30 (or decannulation if earlier). Clinical parameters were reviewed to determine if the patient met the established criteria for PVAP by NHSN criteria^3^ or our proposed criteria for EAP.

We compared these criteria against the standard of culture-confirmed, clinically treated, and independently adjudicated clinical pneumonia (CP). Sensitivity and standard deviation were calculated for each set of diagnostic criteria. To assess the diagnostic accuracy of our criteria, we utilized receiver operating characteristic (ROC) analysis, computing the area under the curve (AUC), and the Mann–Whitney statistic to quantify the strength of association between predicted probabilities and actual outcomes.

## Results

We identified 20 patients representing 505 ECMO days. Our population was 65% male, mean age 46.3 years. Most (85%) received VV ECMO. ECMO indications included COVID ARDS (50%), trauma or inhalational injury (25%), cardiac (20%), non-COVID ARDS (5%). BAL culture organisms included *S. aureus* (45%), *Pseudomonas* species (40%), *Klebsiella* species (15%), and single episodes of other typical hospital-acquired pneumonia organisms.

**Figure 1.**
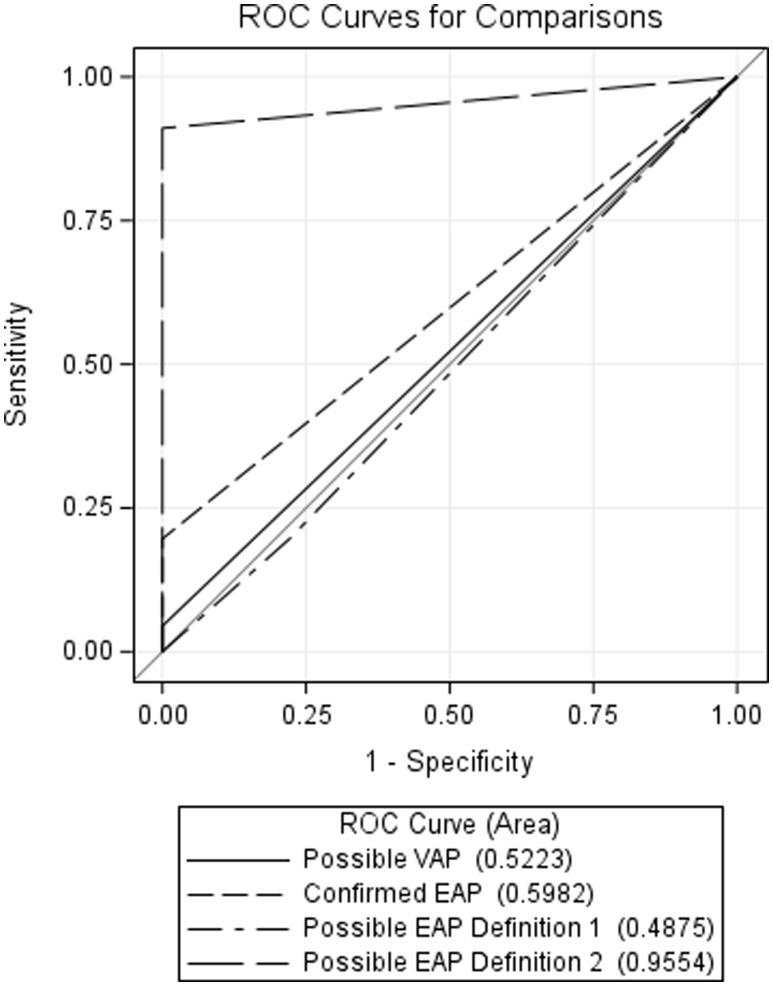
Receiver operating characteristic analysis of the proposed diagnostic criteria for pneumonia to a clinically defined pneumonia cohort.

In 505 ECMO patient days, there were 25 CP events accounting for 112 days. Patients with CP only met criteria for PVAP 1% of the time (Figure 1), a sensitivity of 4.5% (AUC 0.52 [CI = 0.50, 0.54]). “Possible EAP – Definition 2” was the most effective model for predicting CP (sensitivity 91.1%, AUC 0.95 [CI = 0.93, 0.98]), followed distantly by the confirmed EAP model (sensitivity 19.6%) and Possible EAP-Definition 1 (19.6%) (Table 1). All 25 unique CP events met possible EAP -Definition 2 at some point during infectious window and 14/25 (56%) included increase in ECMO parameters.

**Table 1.** Sensitivity of EAP diagnostic criteria. Number of days with pneumonia calculated out of 505 total ECMO days studied.

Diagnostic Criteria	# Days with Pneumonia	% Days with Pneumonia	Sensitivity
Adjudicated Culture Confirmed Pneumonia	112	22%	Proposed standard
Possible VAP	5	1%	4.5%
Possible EAP Definition 1	109	22%	19.6%
Possible EAP Definition 2	102	20%	91.1%
Confirmed EAP	22	4%	19.6%

The Mann–Whitney statistic suggests that the EAP model has a stronger relationship with true outcomes than the PVAP model. Furthermore, contrast estimation and testing results suggests that the estimated difference in AUC between EAP and PVAP models was 0.07 (standard error 0.02). The chi-square test for the contrast estimate was highly significant (*P* < 0.0001).

## Discussion

This study suggests NHSN PVAP criteria are poor predictors of pneumonia in patients on ECMO. This is an important shift in our understanding, as previous research in this field has relied on NHSN PVAP criteria to study epidemiology and outcomes. We are the first group to propose incorporation of ECMO oxygenation parameters (flow and sweep) with infectious parameters and respiratory cultures – thresholds used in critically ill and mechanically ventilated patients. Moreover, we applied this to a population that was predominantly VV ECMO, the group traditionally considered most difficult to study these outcomes. We found this model to be sensitive in predicting pneumonia that was clinically treated in this population.

Without guidelines or definitions, we are unable to identify risk factors that may help prevent pneumonia in ECMO. Our criteria had a strong correlation with cases of pneumonia that were clinically diagnosed and treated. Although our criteria relied on culture data, which limits utility as a preventive tool, we must begin with reliable measures of disease state to understand the true incidence and risk factors.

This study is limited by the small sample size of highly complex patients chosen because of the outcome of interest. There is risk for confounding, particularly given many patients were on ECMO for a prolonged duration, likely due to the coinciding COVID-19 pandemic. We defined our infectious period beginning with time of confirmed infection to best capture infectious events, but further exploration is warranted to understand if predictive measures could be identified prior to infectious outcome. We believe our study supports the utility of modifying current definitions for VAP within critically ill patients by incorporating ECMO-specific parameters. Our work confirms the need for further study to demonstrate the viability of this criterion in a larger and more diverse ECMO cohort.
